# Outcome of patients treated for myelodysplastic syndromes without deletion 5q after failure of lenalidomide therapy

**DOI:** 10.18632/oncotarget.15200

**Published:** 2017-02-08

**Authors:** Thomas Prebet, Andrea Toma, Thomas Cluzeau, Mikkael A. Sekeres, Norbert Vey, Sophie Park, Najla Al Ali, Marie M. Sugrue, Rami Komrokji, Pierre Fenaux, Steven D. Gore

**Affiliations:** ^1^ Section of Hematology, Department of Internal Medicine, Yale University, New Haven, CT, USA; ^2^ Groupe Francophone des Myelodysplasies, Hopital Saint Louis, Paris, France; ^3^ Department of Hematology, Moffitt Cancer Center, Tampa, FL, USA; ^4^ Leukemia Program, Cleveland Clinic, Cleveland, OH, USA; ^5^ Departement d’hematologie, Institut Paoli-Calmettes, Marseille, France; ^6^ Clinique Universitaire d’hématologie, CHU Grenoble Alpes, Grenoble, France; ^7^ Service Hematologie Senior, Hopital Saint Louis, Paris, France; ^8^ Celgene Corporation, Summit, NJ, USA; ^9^ Service d’Hematologie, Hopital Cochin, Paris, France

**Keywords:** myelodysplasia, outcome, lenalidomide

## Abstract

Anemia is a key survival prognostic factor in lower-risk myelodysplastic syndromes (MDS). Lenalidomide (LEN) can correct anemia in 25% of MDS patients without deletion 5q (del5q). As this therapy will inevitably fail, understanding the outcome of these patients will facilitate development of subsequent treatment strategies. To answer this question, an international retrospective study focused on LEN-treated lower-risk, non-del5q, MDS patients was performed. We analyzed the overall survival after LEN failure, its prognostic factors and the impact of post LEN treatment options. We included a total of 384 patients. The median overall survival after failure of LEN was 43 months. In multivariate analysis, adverse cytogenetics, excess of blasts at the initiation of LEN, and the type of failure (classified as stable disease, relapse, intolerance, or progression) were the main determinants of outcome. Subsequent therapy with hypomethylating agents was associated with a prolonged survival compared to BSC (median OS= 51m vs. 36m, *p*=0.01). In conclusion, the survival for non-del5q MDS patients after failure of LEN remains relatively prolonged, though with a wide range. Clinical trial participation remains the recommendation for these patients even if options such as hypomethylating agents may be considered.

## INTRODUCTION

Management of anemia is one of the major challenges for the physician treating lower-risk myelodysplastic syndromes [1]. Depth of anemia and transfusion dependency are associated with an increased risk of mortality in lower-risk MDS. Consequently, hemoglobin level constitutes a key component of current prognostic scoring systems [2, 3]. Transfusion is the mainstay of treatment and to date only limited other options are available. Use of erythropoiesis stimulating agents (ESAs) is the frontline treatment recommended by most guidelines for patients with a low transfusion burden and lower endogenous serum erythropoietin levels. Response rates ranges between 20 and 60% and the median response duration ranges between 12 and 24 months [4]. ESAs improve quality of life but have not yet demonstrated prospectively any improvement of overall survival. In North America, FDA has approved azacitidine for all types of MDS based on CALGB studies but approval is restricted to higher-risk MDS in most of the other countries [5–7]. Several studies, both retrospective and prospective, had been focused on lower-risk MDS treated with hypomethylating agents and showed an overall response rate of 25 to 35% with an expected duration of response of 12 to 18 months [8–10].

Lenalidomide (LEN) has also demonstrated a significant activity in lower-risk MDS [11, 12]. The striking results of lenalidomide in patients harboring deletion 5q quickly led to an approval for this subgroup by FDA and more recently by European agencies. For patients without deletion 5q, efficacy is modest with erythroid response rates between 25% and 27% and a response duration of 32 to 41 weeks [13]. Several international groups have or are evaluating combinations of LEN and ESAs [14, 15]. Clinical activity of the combination seems promising and will warrant further investigation. This synergy may be potentially explained by the capacity of LEN to stabilize the erythropoietin receptor at the membrane [16]. As response rates remain low and duration of response is limited, other agents are currently developed including oral azacitidine [17], telomerase inhibitors [18], and TGF beta inhibitors [19, 20]. Patients previously treated with LEN represent a significant number of the patients included in these ongoing and future trials.

As the development of these new agents reaches phase II and phase III, we need to define the outcome, prognostic factors, and efficacy of current approaches in the population of patients following LEN failure. Using a network of centers in US and Europe, we designed a retrospective study to address these questions.

## PATIENTS AND METHODS

### Patient selection

This study is an international retrospective study including patients from clinical trials [12, 21], compassionate access program, and local registries. Patients from registries were consecutive patients treated in the center/network. Patients were eligible for the analysis if they fulfilled the following criteria: 1/ diagnosis of MDS according to WHO 2008 classification [22], including therapy related MDS 2/ Absence of a deletion 5q confirmed by conventional cytogenetics or fluorescent *in situ* hybridization (FISH) techniques 3/ Lower-risk MDS according to IPSS [23] 4/ treatment with single agent lenalidomide for MDS. All patients gave signed informed consent for the use of their clinical and biological data and Yale University internal review board approved the study.

Patients treated with combinations of lenalidomide and other disease-altering treatments (chemotherapy, hypomethylating agents (HMAs)) were excluded from the analysis. However, combination therapy with ESAs or iron chelation therapies were accepted. Patients treated with lenalidomide for remission maintenance, for instance after allogeneic transplantation, were also excluded from the analysis.

Cytogenetic risk was assessed based on IPSS [23]. RBC transfusion dependency was defined by the need of at least 4 units of RBC over an 8 weeks’ period before the initiation of therapy.

### Definition of lenalidomide response and resistance

Clinical and cytogenetic responses were evaluated according to the international working group 2006 MDS criteria [24]. We defined 4 different categories: absence of response, bone marrow progression during treatment with or without prior response, secondary failure (loss of a prior hematological response without bone marrow progression), and intolerance (treatment discontinuation due to adverse event, with or without prior response).

### Statistical methods

Data were summarized by frequency and percentage for categorical variables. For continuous variables, the median and range were computed. All results are presented with their 95% confidence intervals. Statistical tests were two-sided at the 5% level of significance. To investigate the association between continuous variables and categorical variables, univariate statistical analyses were performed using non-parametric Wilcoxon rank sum test, Chi square test or Fisher's exact test when appropriate. Survival rates were estimated by the Kaplan-Meier method. Overall survival (OS) was measured from the date of LEN failure until death from any cause with observation ending at the date of last contact for patient last known to be alive. Leukemia Free Survival was measured from the time of initiation of LEN to the time of documentation of AML, death from other causes being here considered as a competitive risk. Patients without events were censored at the date of last follow up. Multivariate analyses were performed using a Cox proportional hazards method. All variables with p-value below 0.15 in univariate analysis were included in the Cox model using a stepwise procedure selection. Statistical tests were performed using SPSS 21.0 and graphs were performed using PRISM 6 software.

## RESULTS

### Patients’ characteristics

A total of 384 eligible patients treated between 2003 and 2015 who met eligibility criteria were included in this study. The accrual between US and Europe was well balanced with respectively 211 and 173 cases. Patients from clinical trials represented 117 cases (30%). Patients’ characteristics are depicted in Table 1. All patients were diagnosed and treated with LEN for lower-risk MDS. IPSS was classified as low risk in 41.4% of the cases (*n* = 159) or intermediate-1 risk in 58.6% of the cases (*n* = 225). The median time between diagnosis and initiation of LEN was 24 months (range [1–115]). Median bone marrow blast percentage was 2% and 69 patients (18%) were classified as RAEB-1. Cytogenetic findings based on IPSS classification were favorable, intermediate, and unfavorable in 81%, 12%, and 6% of cases respectively, including 274 (71%) with normal karyotypes. Fifty-five percent of the patients (*n* = 211) received ESA and 23% (*n* = 89) received HMA before the initiation of LEN. Supplemental Table 1 shows the patients’ characteristics based on prior MDS therapies. Of note, 92% of the patients were RBC transfusion dependent at the initiation of LEN therapy.

**Table 1:** Patient characteristics

Variable	
**N**=	384
**Median age**	71 (23-89)
**Sex Ratio M/F**	258/126
**therapy-related MDS**	34 (8%)
**WHO classification**	
RA/RARS/MDS-U	31 (8%) /124 (33%) /24 (6%)
RCMD/ RCMD-RS	136 (36%)
RAEB-1	69 (18%)
**Median BM blast %**	2 (0-9)
**IPSS Cytogenetic stratification**	
Favorable	310 (81%) (274 normal K)
Intermediate	47 (12%)
Unfavorable	23 (6%)
Unknown	4 (1%)
**RBC TD before LEN**	354 (92%)
**Use of ESA before LEN**	211 (55%)
**Use of HMA before LEN**	89 (23%)
**LEN response**	78 (20%)
**LEN duration (months)**	4m (1-63)

M/F: male/female, RA: refractory anemia, RARS: refractory anemia with ring sideroblasts, MDS-U: myelodysplastic syndrome of unknown classification, RCMD: Refractory cytopenia with multilineage dysplasia, RAEB: Refractory anemia with excess of blast, BM: bone marrow, K.: karyotype, RBC: Red blood cell, TD:transfusion dependency, LEN: lenalidomide, ESA: erythropoiesis stimulating agent, HMA: hypomethylating agents, m: month.

Most patients were treated with LEN 10mg/d continuously (*n* = 174), 10mg for three weeks followed by a one week off (*n* = 90), or received 5 mg/d continuously (*n* = 116) as published [11, 25]. A total of 264 patients (65%) received 10mg/d at the initiation of therapy. The median duration of LEN therapy was 4 months (range: [1–63]) and 20% of the patients (*n* = 78) had an erythroid response according to 2006 IWG criteria. Concomitant treatment with ESA+LEN was documented in a minority of patients (*n* = 45). At the time of LEN failure, 64% of the patients had never achieved response but did maintained stable disease (*n* = 245, median duration of LEN of 4 months), 17% of patients had lost their HI after an initial response (*n* = 67, median duration of LEN of 15 months), and 12% of the patients stopped LEN for intolerance (*n*= 40, median duration of LEN of 3 months). Disease progression to RAEB-2 or AML was documented in an additional 9% of patients (*n* = 33, median duration of LEN of 3 months).

### Outcome of patients after lenalidomide failure

**Figure 1:**
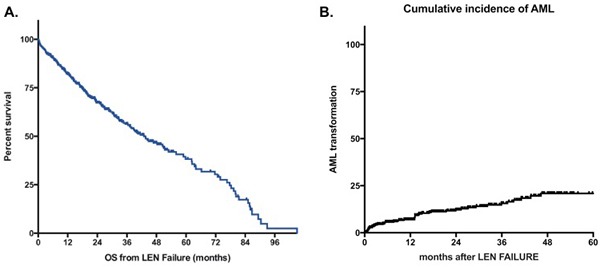
A Overall Survival of MDS patients without deletion 5q after lenalidomide failure**. B**. Cumulative incidence of AML. Survival is defined from documentation of failure to death of any cause or last-follow-up and is expressed in months. LEN: lenalidomide.

**Figure 2:**
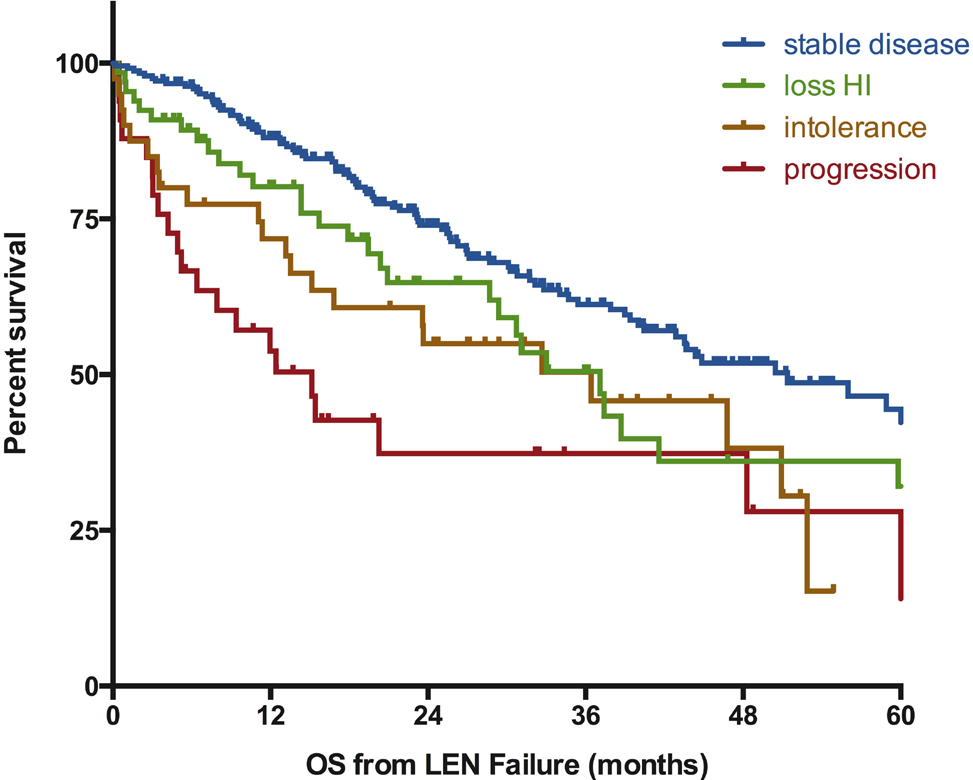
Influence of the type of lenalidomide failure on the outcome of MDS patients without deletion 5q Survival is defined from documentation of failure to death of any cause or last-follow-up and is expressed in months. SD: stable disease, loss of HI: loss of hematologic improvement without bone marrow progression, PD: progressive disease at failure (to RAEB-2 or AML), LEN: lenalidomide.

At last follow-up, 203 patients were alive and 181 patients had died. The median follow-up after LEN failure for surviving patients was 24 months. For patients with ESA failure and RBC transfusion dependency at the initiation of LEN, the median survival from LEN initiation was 45 months (95% CI [38–56]). The estimated median overall survival of the whole cohort after failure of LEN was 43 months (95%CI [35–51]) (Figure 1A). For patients with persistent lower-risk disease at the time of failure of LEN, the risk of progression to leukemia was low with a probability of 12% at 2 years and 21% at 5 years (Figure 1B). Overall survival of patients without response to LEN was 51 months (95%Ci [39–64]) (Figure 2). OS for patients with loss of erythroid response or intolerance to LEN were 37m (95%CI [29–45]) and 36 months (95%CI [15–57]) respectively. Patients with disease progression had a dismal outcome with a median OS of 15 months (95%CI [8–22]).

In univariate analyses, several factors had a negative impact on survival: presence of excess of blasts at initiation of LEN (24m for RAEB-1 *vs*. 45m for others, *p* < 0.001), presence of an unfavorable karyotype (17m with *vs*. 44m without, *p* = 0.007), and type of treatment failure (see above, *p* = 0.001). Several other factors had also a borderline significant association with survival: Age with a cut-off value at 60y (74m below 60y *vs*. 43m above, *p*= 0.09), History of t-MDS (27m with *vs*. 44m without, *p* = 0.09), absence of response to LEN (51m without *vs*. 37m with, *p* = 0.06), and prior treatment with HMAs (35m with *vs*. 45m without, *p* = 0.14). Of note, the subgroup of patients with RARS (*n* = 125) had a median OS of 47 months as compared to 43 months for non-RARS patients (*p* = 0.16). There was no prognostic impact of region (Europe *vs*. US), sex, prior treatment with ESA, IPSS (low *vs*. intermediate-1), dose of LEN, duration of LEN, concomitant treatment with ESA, or response to LEN. Of note, the prognostic value of R-IPSS cytogenetics could not be evaluated, as the number of patients in very good and very poor groups were too small to be analyzed.

The multivariate model (Table 2) included all of the above mentioned variables with an impact on OS. It showed that presence of excess of blasts at the initiation of LEN (HR 1.60 95%CI [1.08-2.4], *p* = 0.018), presence of unfavorable cytogenetics (HR 1.63 95%Ci [0.97-2.73], *p* = 0.066), and type of failure (as compared to SD: loss of HI (1.43 [0.96-2.14], *p* = 0.073; intolerance (1.92 [1.20-3.08], *p* = 0.007), progression (2.12 [1.30-3.45], *p* = 0.003)) retained their prognostic values.

**Table 2:** Multivariate analysis of outcome after lenalidomide failure

Variable	Median OS	HR	95%CI	*P* value
Adverse K no Adverse K yes	44m 17m	1 1.63	[0.95-2.42]	0.066
RAEB no RAEB yes	45m 24m	1 1.51	[1.08 - 2.35]	**0.03**
SD Loss HI Intolerance PD at failure	51m 37m 36m 15m	1 1.44 1.92 2.12	[0.97-2.14] [1.20-3.08] [1.30-3.47]	0.07 **0.007** **0.003**
Age 60y or less More than 60y	74m 43m	1 1.48	[0.90-2.42]	0.12
No t-MDS t-MDS	44m 27m	1 1.51	[0.91-2.49]	0.11
No prior HMA HMA	45m 35m	1 1.18	[0.83-1.67]	0.37

OS: overall survival, HR: Hazard ratio, CI: confidence interval, y: year, m: month, adverse K: adverse karyotype (per IPSS classification), RAEB: refractory anemia with excess of blasts (here limited to 5% to 10% bone marrow blasts), SD: stable disease, loss of HI: loss of hematologic improvement without bone marrow progression, PD: progressive disease at failure (to RAEB-2 or AML), LEN: lenalidomide, t-MDS: therapy-related MDS.

### Impact of treatment strategies after failure of lenalidomide

**Figure 3:**
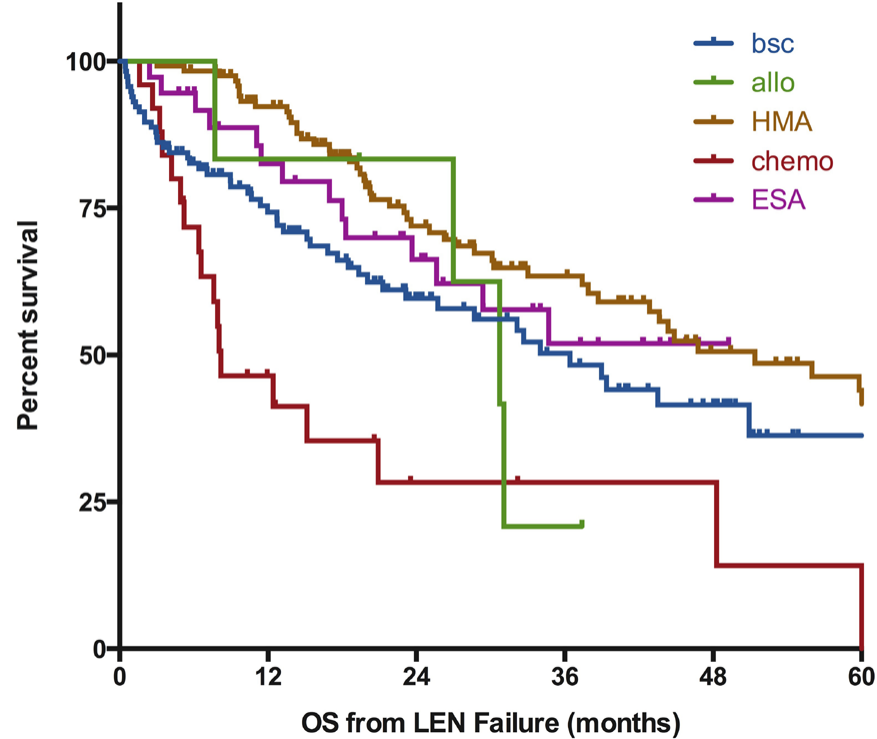
Impact of post-lenalidomide strategies on outcome of MDS patients without deletion 5q Survival is defined from documentation of failure to death of any cause or last-follow-up and is expressed in months. BSC; best supportive care, chemo: chemotherapy (including AML like induction regimen or lower dose standard chemo), HMA: hypomethylating agents, ESA: erythropoiesis stimulating agents, Allo: allogeneic transplantation, LEN: lenalidomide.

Information on the treatment given after failure of LEN was available for 358 patients (93% of the cohort). Their characteristics are depicted in Supplemental Table 2. Best supportive care was the only modality used for 116 patients (32%), who had a median overall survival of 36 months (95%CI [26–47], as seen on Figure 3). Receiving other MDS therapies was associated with a significantly better survival (median OS 44 months 95%CI [35–54], *p* = 0.03). Of note, we did not observe a significant benefit of active treatment in the subgroup of patients with RARS at the time of failure (median OS 51 months for patients treated with BSC (*n* = 53) *vs* 62 months for patients treated with active treatment (*n* = 55), *p* = 0.52, see Supplemental Figure 1)

It is important to notice that outcome varied according to the strategy: a group of patients (*n* = 123, 34%) was treated with HMAs (74% with azacitidine), with only 11 of them treated for progressive disease. Thirty patients (of 115 patients with available response data, 26%) were documented as responders and median survival was 51 months (95%CI [35–67], *p* = 0.01 as compared to BSC). Of note, the response rate to HMA was not statistically different between the different types of failure (28% for non-responders *vs* 20% for the other patients, *p* = 0.33). Response to HMA in patients with RARS was 36% (9/25 evaluated patients, *p* = 0.2 *vs* non-RARS patients (23%, 21/90)). Thirty-seven patients (10%) received ESA, including only 3 patients not previously treated with ESA before LEN. 1 response was noted out of 22 reported outcomes; median OS was not reached (*p* = NS as compared to BSC), likely reflecting selection of a patient group with indolent disease (Supplemental Table 2).

Response to conventional chemotherapy for the 25 patients experiencing disease progression was 20% (5 CR); median survival was 8 months (95%CI [3–14], *p* < 0.001 as compared to BSC). Allogeneic transplantation was the upfront treatment after LEN failure for only 6 patients; an additional 19 patients were allotransplanted after other modalities of treatment. The median OS of these 25 patients was 43 months (*p* = 0.84 as compared to BSC). Among the remaining 51 patients treated with other modalities, 12 patients received immunosuppressive therapies (ATG +/- cyclosporine (*n* = 6), cyclosporine alone (*n* = 3), rituximab (*n* = 2), TNF inhibitor (*n* = 1), 3 responders), 9 patients received thalidomide (2 responders), 30 patients received investigational agents (ruxolitinib, erlotinib, rigosertib, TLK199, TGF beta inhibitors, and others). 4 of these 51 patients responded to treatment.

## DISCUSSION

This is the first large study focusing on the outcome of patients with lower-risk myelodysplastic syndromes without deletion 5q after failure of lenalidomide. We observed a good overall survival of the patients with a median of 43 months and showed that disease characteristics and type of failure were the main determinants of outcome after failure of LEN. Hypomethylating agents may represent one of the suitable options for these patients.

Our data are consistent with the literature for patients experiencing ESA failure [26, 27] with a published OS of 40 to 45 months in this group of patients. We observed a median survival of 45 months from LEN initiation for ESA treated patients (as compared to 54 months for ESA naïve patients, *p* = NS, see Supplemental Table 3 for details). The relatively low percentage of patients exposed to ESA (55%) can be explained by the regional differences of availability and prescription of ESA and LEN between Europe and North America. ESA treatment (prior to LEN or concomitantly) did not seem to influence outcome after LEN failure. However, we did not have enough data on ESA treatment to confirm the recently reported poorer prognosis of early ESA failure (within 6 months of ESA onset) [26]. it is also important to stress that our goal was not to comment or analyze specifically the results of LEN in lower-risk MDS, our cohort being defined by the failure of the therapy. Nevertheless, we observed a response rate and a duration of response in line with the literature.

Not surprisingly, excess of blasts and IPSS cytogenetic risk both influenced outcome after LEN failure. Similarly, survival for patients with stable disease was greater than the survival of patients experiencing progression to RAEB-2 or AML. What was more surprising was the trend to a better survival for patients without response to LEN as compared to patients experiencing at least an HI. This may reflect the relative dissociation between response and survival in this group of patients and is consistent with the “compensation” of this difference of overall survival when survival was calculated from LEN initiation (55m for responders *vs* 48m for non-responders respectively, *p* = 0.05). It suggests that LEN does not really alter the natural history of the disease in this group of patients and, once again, this finding will be important to take into consideration in the design of future trials. Some variables such as mutations [28, 29], co-morbidities, or endogenous erythropoietin levels were only available in a proportion of patients lower than 20% and we were not able to integrate them in the prognosis model. Follow-up studies integrating these variables will be useful to refine our prognosis model.

Most international guidelines [30, 31] favor the use of ESA as frontline treatment for lower-risk MDS patients with anemia, even though MDS-associated anemia is not yet a specific labeled indication by FDA or EMA. For second line treatment, the only registered treatment option is HMA in North America. Lenalidomide is not registered for second line treatment of non-del5q MDS patients and results of LEN monotherapy are relatively modest in an unselected population. As previously mentioned, more promising results have been showed with combination of LEN and ESA [14, 15] and several groups are currently trying to define subgroups of patients more susceptible to the drug [13, 15, 32]. This let us think that a significant proportion of non-del5q MDS patients will still be treated in the next years with this agent, in particular in the context of clinical trials.

Regarding treatment options, our data and one smaller prior study [33], suggest that in non-del5q patients, there may be a survival benefit of using sequentially LEN before HMA for lower-risk patients. These data need to be interpreted with caution, as a selection bias for which patients received HMAs may have contributed to this outcome (Supplemental Table 2). As we and other had shown that survival of lower-risk MDS is prolonged, the treatment decision should also integrate variable such as co-morbidities and quality of life. From this perspective, hypomethylating agents treatment may represent a challenge for the more frail and older population. New investigational agents including oral AZA, TGF beta Inhibitors [19, 20] or Imetelstat [18], may impact treatment strategies, including the use of TGF beta inhibitors for patients with RARS [20]. Some of these trials exclude patients previously treated with LEN and we will need to have additional data to be able to clearly define the best sequences and treatment algorithms for our patients, acknowledging that, even if survival is prolonged, cure is still not achievable for the vast majority of them. Finally, this relatively long overall survival also stresses the challenge that will represent any clinical investigation using overall survival as a primary endpoint in this group of patients.

## SUPPLEMENTARY MATERIALS FIGURES AND TABLES


