# Supplementary material for: Outcome of patients treated for myelodysplastic syndromes without deletion 5q after failure of lenalidomide therapy
Source: Oncotarget. 2017 Feb 8;8(23):37866–74. doi: 10.18632/oncotarget.15200 (PMC5514957; doi:10.18632/oncotarget.15200)
Supplement: Supplementary file 1 [file oncotarget-08-37866-s001.pdf]

Outcome of patients treated for myelodysplastic syndromes without deletion 5q after failure of lenalidomide therapy

Supplementary Material

Supplemental Table 1: Details of patients' characteristics based on prior MDS therapies

BSC: best supportive care, chemo: chemotherapy (including AML like induction regimen or lower dose standard chemo), HMA: hypomethylating agents, ESA: erythropoiesis stimulating agents.

**Variable**	Whole cohort	No prior HMA or ESA	Prior ESA and no HMA	Prior HMA and no ESA	Prior ESA and HMA
**N=**	384	138	157	35	54
**Median age**	71 (23-89)	70 (27-87)	72 (47-89)	74 (57-80)	68 (23-80)
**Male gender**	258 (67%)	94 (68%)	108 (69%)	21 (60%)	32 (59%)
**Therapy related MDS**	34 (8%)	18 (13%)	7 (4%)	1 (3%)	8 (15%)
**WHO classification**					
RAEB-1	69 (18%)	21 (15%)	25 (16%)	12 (34%)	11 (20%)
**Median BM blast %**	2 (0-9)	2 (0-9)	2 (0-9)	4 (1-9)	2 (0-9)
**IPSS Cytogenetic stratification**					
Favorable	310 (81%)	106 (76%)	135 (86%)	25 (71%)	44 (81%)
Intermediate	47 (12%)	18 (13%)	19 (12%)	4 (11%)	6 (11%)
Unfavorable	23 (6%)	13 (9%)	1 (1%)	5 (14%)	4 (7%)
Unknown	4 (1%)	1 (1%)	2 (1%)	1 (3%)	0
**RBC TD before LEN**	354 (92%)	130 (94%)	144 (92%)	34 (97%)	46 (85%)
**LEN response**	78 (20%)	34 (25%)	29 (18%)	7 (20%)	8 (15%)
**LEN duration (months)**	4m (1-63)	4 (1-54)	4 (1-63)	3 (1-38)	4 (1-17)

Supplemental Table 2: Details of patients' characteristics in each treatment group.

BSC: best supportive care, chemo: chemotherapy (including AML like induction regimen or lower dose standard chemo), HMA: hypomethylating agents, ESA: erythropoiesis stimulating agents.

Variable	BSC	HMA	ESA	Chemo
**N=**	116	123	37	25
**Median age**	73 (54-89)	68 (32-87)	72 (41-85)	71 (54-83)
**Male gender**	79 (68%)	86 (70%)	22 (59%)	14 (56%)
**Therapy related MDS**	12 (10%)	11 (9%)	2 (5%)	2 (8%)
**WHO classification**				
RAEB-1	26 (22%)	18 (15%)	10 (27%)	13(51%)
**Median BM blast %**	2 (0-9)	2 (0-9)	3 (0-9)	4 (0-9)
**IPSS Cytogenetic stratification**				
Favorable	96 (84%)	97 (79%)	31 (84%)	18 (72%)
Intermediate	16 (14%)	16 (13%)	6 (16%)	1 (4%)
Unfavorable	3 (3%)	10 (8%)	0	6 (24%)
Unknown	1 (1%)	0	0	0
**RBC TD before LEN**	109 (94%)	119 (97%)	30 (81%)	21 (84%)
**Use of ESA before LEN**	101 (87%)	36 (29%)	34 (92%)	11(44%)
**Use of HMA before LEN**	20 (17%)	20 (16%)	9 (24%)	16 (64%)
**LEN response**	20 (17%)	24 (20%)	3 (8%)	5 (20%)
**LEN duration (months)**	4 (1-63)	4 (1-52)	4 (1-19)	3 (1-38)
**Progression after LEN**	9 (8%)	11 (9%)	0	25 (100%)

Supplementary Table 3: Survival estimates based on prior MDS therapies.
Median overall survival (OS) is expressed in months. HMA: hypomethylating agents, ESA: erythropoiesis stimulating agents.

	OS from diagnosis	OS from LEN initiation	OS from LEN failure
ESA naïve patients	103 months	54 months	50 months
ESA pretreated patients	88 months	45 months	40 months
p=	0.02	0.46	0.3
HMA naïve patients	93 months	54 months	45 months
HMA pretreated patients	92 months	40 months	35 months
p=	0.58	0.12	0.14

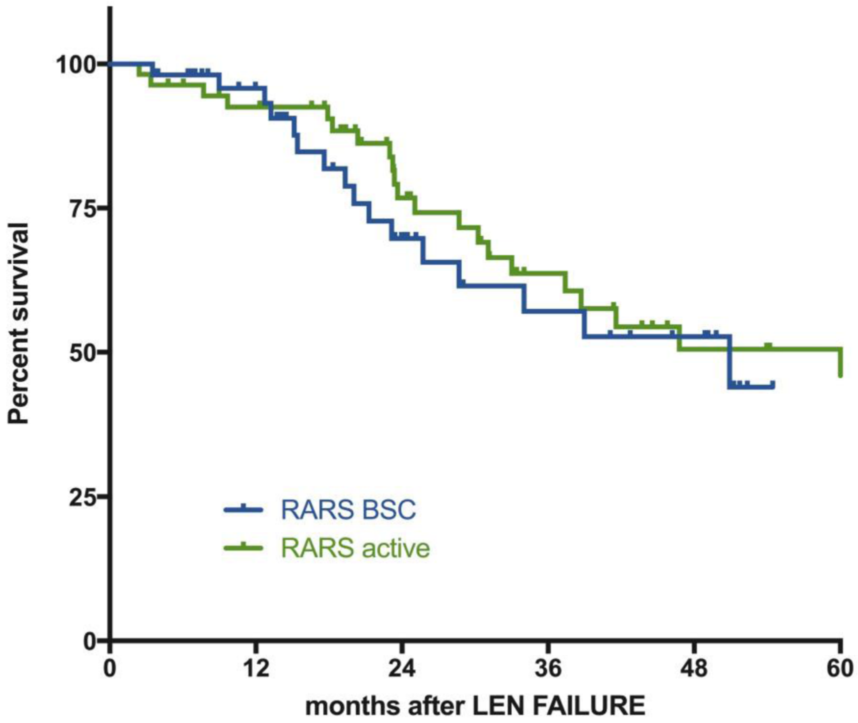

Supplemental Figure 1: Impact of active treatment on outcome of RARS patients experiencing lenalidomide (LEN) failure.

Kaplan Meier estimate of survival with time expressed in months. Survival is defined from date of LEN failure to death or last follow-up. Each tick-mark represents a censored patient. Patients with progression at the time of LEN failure were excluded from the analysis. BSC: best supportive care, Active: active treatment including ESA, HMA, clinical trials.
